# Cardiometabolic Benefits and Risks of Sodium-Glucose Co-Transporter-2 (SGLT-2) Inhibitor and Glucagon-Like Peptide-1 (GLP-1) Receptor Agonist Combination Therapy in Type 2 Diabetes: A Systematic Review

**DOI:** 10.7759/cureus.96218

**Published:** 2025-11-06

**Authors:** Dalia Tantawy, Maram Rabih Musa Rabih, Razan Seifeldin Ibrahim Mohamed, Malaz Omer Abdelrahman Ali, Rayan Saad Aldeen Mohammed Saad Aldeen, Ali Omer Ahmed Abdelkarim, Tawasul Greeballah Awadalkreem Mohamedzain

**Affiliations:** 1 General Medicine, George Eliot Hospital, West Midlands, GBR; 2 Internal Medicine, National University Sudan, Khartoum, SDN; 3 Psychiatry, Royal College of Surgeons in Ireland, Dublin, IRL; 4 General Medicine, Noble's Hospital, Braddan, IMN; 5 Internal Medicine, Faculty of Medicine, Igraa College for Science and Technology, Gezira State, SDN; 6 Internal Medicine, Hayat National Hospital, Qassim, SAU; 7 Internal Medicine, Umluj General Hospital, Umluj, SAU

**Keywords:** cardiovascular outcomes, combination therapy, glp-1 receptor agonist, sglt2 inhibitor, systematic review, type 2 diabetes

## Abstract

Type 2 diabetes (T2DM) management increasingly focuses on cardiometabolic protection. Sodium-glucose co-transporter-2 inhibitors (SGLT2is) and glucagon-like peptide-1 receptor agonists (GLP-1 RAs) have complementary mechanisms, making their combination a promising strategy. This systematic review evaluates the cardiometabolic benefits and risks of SGLT2i and GLP-1 RA combination therapy in T2DM. Following Preferred Reporting Items for Systematic Reviews and Meta-Analyses (PRISMA) guidelines, a systematic search of PubMed, Scopus, Embase, and Web of Science was conducted for randomised controlled trials (RCTs) published between 2020 and 2025. Nine RCTs, including cardiovascular outcome trials and mechanistic studies, were included. Data on cardiovascular, renal, glycaemic, and safety outcomes were extracted. The Cochrane RoB 2 tool was used for quality assessment. Combination therapy demonstrated additive cardioprotective effects. Post-hoc analyses showed GLP-1 RAs reduced major adverse cardiovascular events (MACE) and heart failure hospitalisation regardless of background SGLT2i use, and SGLT2i benefits were maintained with concomitant GLP-1 RAs. The combination provided superior glycaemic control (HbA1c reduction), weight loss, and systolic blood pressure reduction compared to monotherapy. The safety profile was consistent with the known effects of each drug class, with no new or unexpected safety signals and manageable gastrointestinal and genital infection risks. The combination of SGLT2is and GLP-1 RAs in T2DM provides synergistic benefits for cardiovascular risk reduction, glycaemic control, weight, and blood pressure, without a significant increase in adverse events. This supports its use as a potent therapeutic strategy for high-risk patients, though definitive evidence from prospective trials designed specifically for combination therapy is still needed.

## Introduction and background

Type 2 diabetes mellitus (T2DM) is a chronic, progressive metabolic disorder characterised by insulin resistance, impaired insulin secretion, and persistent hyperglycemia [1]. It affects over 500 million individuals worldwide and remains a leading cause of cardiovascular morbidity and mortality [2]. Beyond elevated glucose levels, T2DM substantially increases the risk of heart failure, chronic kidney disease (CKD), and atherothrombotic events. Consequently, diabetes management has shifted from a glucose-centric approach to one emphasising comprehensive cardiometabolic protection, addressing both metabolic control and cardiovascular risk [3].

Among contemporary therapies, two novel drug classes, sodium-glucose co-transporter 2 inhibitors (SGLT-2i) and glucagon-like peptide-1 receptor agonists (GLP-1 RA), have demonstrated significant cardiovascular and renal benefits that extend beyond glycaemic reduction [4]. SGLT-2i promotes urinary glucose (glucosuria) and sodium (natriuresis) excretion, resulting in lower plasma glucose, body weight, and blood pressure, while improving heart failure outcomes [5]. In contrast, GLP-1 RAs stimulate glucose-dependent insulin secretion, suppress glucagon release, delay gastric emptying, and enhance satiety, leading to improved glycaemic control and weight reduction [6]. Landmark cardiovascular outcome trials (CVOTs) such as EMPA-REG OUTCOME (empagliflozin), CANVAS (canagliflozin), and LEADER (liraglutide) demonstrated reductions in major adverse cardiovascular events (MACE), establishing these therapies as cornerstones of cardiometabolic risk mitigation in T2DM [7].

Given their complementary mechanisms and distinct organ benefits, combination therapy with an SGLT-2 inhibitor and a GLP-1 receptor agonist has been proposed as a strategy to optimise metabolic and cardiovascular outcomes [4]. Theoretically, this dual approach could yield additive improvements in glycaemic control, weight loss, and blood pressure reduction, potentially amplifying protection against cardiovascular and renal complications [8]. However, much of the current evidence supporting such benefits is derived from post-hoc or subgroup analyses rather than prospective randomised combination trials. Furthermore, concerns persist regarding safety, tolerability, cost-effectiveness, and adverse events such as genital infections, gastrointestinal disturbances, and volume depletion. Clinical data on long-term outcomes and comparative efficacy remain limited and heterogeneous [9].

Therefore, this systematic review aims to critically evaluate the existing evidence on the efficacy and safety of combined SGLT-2 inhibitor and GLP-1 receptor agonist therapy in adults with type 2 diabetes, with particular focus on its effects on glycaemic control, cardiovascular outcomes, renal function, and metabolic risk factors.

## Review

Methodology

Review Protocol

This systematic review was conducted in accordance with the Preferred Reporting Items for Systematic Reviews and Meta-Analyses (PRISMA 2020) guidelines [10]. The review protocol was developed prior to the initiation of the study to ensure methodological transparency and consistency.

Eligibility Criteria

The eligibility criteria were defined using the Population, Intervention, Comparison, Outcomes, and Study Design (PICOS) framework, as summarised in Table 1. Only randomised controlled trials (RCTs) published between January 2020 and October 2025 were included to capture the most recent and clinically relevant evidence regarding the cardiometabolic benefits and risks of combination therapy with SGLT-2 inhibitors and GLP-1 receptor agonists in type 2 diabetes. The decision to include RCTs exclusively was based on their robust methodological design, which minimises bias and allows reliable assessment of causality between interventions and outcomes. Non-randomised studies, observational analyses, reviews, case reports, and conference abstracts were excluded to ensure data quality and comparability.

**Table 1:** Eligibility criteria (PICOS framework)

Parameter	Description
Population (P)	Adults (≥18 years) diagnosed with Type 2 Diabetes Mellitus (T2DM).
Intervention (I)	Combination therapy involving an SGLT-2 inhibitor and a GLP-1 receptor agonist.
Comparison (C)	Monotherapy with either SGLT-2 inhibitor or GLP-1 receptor agonist, or placebo.
Outcomes (O)	Primary: Glycemic control (HbA1c), cardiovascular outcomes, and renal outcomes. Secondary: Body weight, blood pressure, lipid profile, and adverse events.
Study Design (S)	Randomized Controlled Trials (RCTs) published from 2020–2025.

Information Sources

An extensive electronic search was conducted across PubMed, Scopus, Embase, and Web of Science to identify eligible studies. The search covered publications from January 2020 to October 2025, without language restrictions. Additional studies were identified through citation searching of relevant review articles and reference lists of included studies to ensure comprehensive literature coverage.

Search Strategy

The search strategy was designed using a combination of Medical Subject Headings (MeSH) and free-text terms relevant to SGLT-2 inhibitors, GLP-1 receptor agonists, and Type 2 Diabetes Mellitus. Boolean operators (AND, OR) and truncations were used to refine the search. The detailed search strategy for PubMed is presented in Table 2, and similar strategies were adapted for other databases.

**Table 2:** Search strategy example (PubMed)

Concept	Keywords/Search Terms
Population	“Type 2 Diabetes Mellitus” OR “T2DM” OR “Diabetes Mellitus, Type 2”
Intervention	“SGLT-2 inhibitor” OR “sodium-glucose cotransporter 2 inhibitors” OR “empagliflozin” OR “dapagliflozin” OR “canagliflozin”
Comparison/Combination	“GLP-1 receptor agonist” OR “glucagon-like peptide-1 receptor agonist” OR “liraglutide” OR “semaglutide” OR “dulaglutide”
Outcomes	“Cardiovascular outcomes” OR “glycemic control” OR “renal outcomes” OR “metabolic outcomes” OR “safety”
Final Search String	(“Type 2 Diabetes Mellitus”) AND (“SGLT-2 inhibitors”) AND (“GLP-1 receptor agonists”) AND (“Randomized Controlled Trial”)

Selection Process

All retrieved records were imported into EndNote X6 for citation management, and duplicates were automatically removed. The remaining records were screened in two phases: title/abstract screening and full-text review. Two independent reviewers performed the screening based on predefined inclusion and exclusion criteria. Discrepancies were resolved through discussion and consensus with a third reviewer to minimise selection bias. The study selection process is illustrated using a PRISMA flow diagram.

Data Collection Process

A standardised data extraction form was used to collect information from each included study. Extracted data included study characteristics (author, year, country, sample size), participant demographics, intervention details (type and dosage of SGLT-2 inhibitor and GLP-1 RA), comparator, duration of follow-up, and reported outcomes (glycaemic, cardiovascular, renal, and safety). Data extraction was performed independently by two reviewers to ensure accuracy, with disagreements resolved by consensus.

Data Items

The primary data items extracted were changes in HbA1c (%), body weight (kg), systolic and diastolic blood pressure (mmHg), major adverse cardiovascular events (MACE), heart failure hospitalisation, and renal outcomes. Secondary outcomes included adverse events such as hypoglycaemia, genital infections, gastrointestinal symptoms, and treatment discontinuation rates.

Study Risk of Bias Assessment

The methodological quality and risk of bias of each included RCT were assessed using the Cochrane Risk-of-Bias tool for Randomised Trials (RoB 2) [11]. This tool evaluates potential biases across five domains: randomisation process, deviations from intended interventions, missing outcome data, measurement of outcomes, and selection of the reported result. Each domain was rated as “low risk”, “some concerns”, or “high risk”, and overall study quality was summarised accordingly.

Synthesis Methods

Due to considerable clinical and methodological heterogeneity across the included RCTs, particularly in terms of intervention duration, participant characteristics, drug combinations, and outcome measures, a meta-analysis was not performed. Instead, a qualitative synthesis of the evidence was undertaken to summarise findings and identify consistent trends across studies. Conducting a meta-analysis in the presence of high heterogeneity could lead to misleading pooled estimates and reduced validity of conclusions.

Results

Study Selection Process

**Figure 1:**
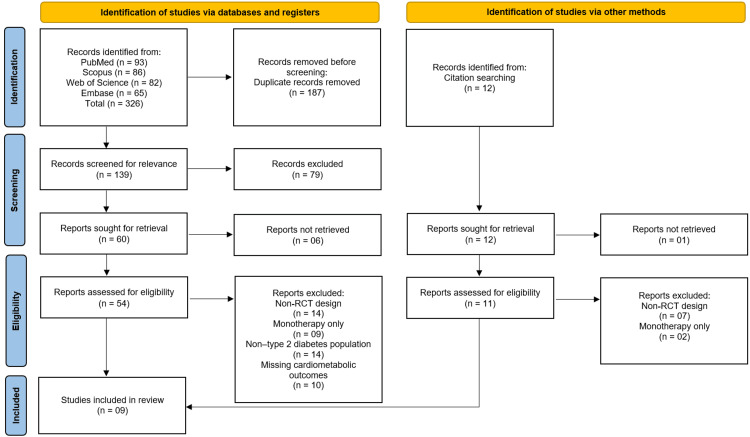
Studies the identification process on the PRISMA flowchart

The systematic search across four electronic databases (PubMed, Scopus, Web of Science, and Embase) initially identified 326 records. After the removal of 187 duplicate records, a total of 139 unique records were screened based on their titles and abstracts. Of these, 79 records were excluded for not meeting the inclusion criteria. The full texts of the remaining 60 reports were sought for retrieval, of which six could not be accessed, leaving 54 reports for full-text eligibility assessment. A further 44 reports were excluded for the following specific reasons: non-randomised controlled trial design (n=14), investigation of monotherapy only and not combination therapy (n=9), study population did not consist of patients with type 2 diabetes (n=14), or missing relevant cardiometabolic outcomes (n=10). An additional 12 records were identified through citation searching of relevant articles. After screening, 11 of these were retrieved and assessed, with nine being excluded (seven for non-RCT design and two for monotherapy only). Ultimately, this process yielded a total of nine studies [12-20] that met all pre-defined eligibility criteria and were included in the systematic review (Figure 1).

Study Characteristics

A total of nine studies [12-20] were included in this systematic review, comprising a mix of large-scale CVOTs, smaller mechanistic studies, and one study protocol for an ongoing trial. The characteristics of these studies are summarised in Table 3. The included studies were published between 2020 and 2024 and involved multinational or specific regional populations. Sample sizes varied widely, from 60 participants in smaller mechanistic investigations [19] to over 17,000 in a large post-hoc analysis [16].

**Table 3:** Characteristics of included randomized controlled trials

Study (Year)	Country / Region	Study Design	Sample Size (n)	Population Characteristics	Intervention (Drug + Dose)	Comparator (Drug + Dose)	Follow-up Duration	Primary Outcome
Neves et al., [12] (2023)	Multinational	Post hoc analysis of RCT	9,462	Adults with type 2 diabetes and established cardiovascular or renal disease; 6.1–8.8% were on background SGLT2 inhibitors	GLP-1 RA: Albiglutide and Efpeglenatide; dose as per original trials	Placebo, with or without background SGLT2 inhibitor therapy	Median 1.5–2 years	Major adverse cardiovascular events (composite of CV death, myocardial infarction, or stroke)
Lam et al., [13] (2022)	Multinational	Randomized, double-blind, placebo-controlled, parallel-group cardiovascular outcome trial	4,076 participants (15.2% on baseline SGLT2 inhibitors, n=618)	Adults with Type 2 Diabetes Mellitus and established cardiovascular or renal disease	Efpeglenatide (GLP-1 receptor agonist), once-weekly subcutaneous injection (dose: 4 mg or 6 mg)	Placebo, once-weekly injection; both groups on standard of care ± SGLT2 inhibitor	Median 1.8 years	MACE — composite of cardiovascular death, nonfatal myocardial infarction, or nonfatal stroke
Jabbour et al., [14] (2020)	Multicenter (International)	Phase 3, multicenter, double-blind, randomized, active-controlled trial	695	Adults (≥18 years) with type 2 diabetes inadequately controlled on stable metformin monotherapy (≥1,500 mg/day); baseline HbA1c 8.0–12.0% (64–108 mmol/mol)	Exenatide 2 mg once weekly (QW) + Dapagliflozin 10 mg once daily	(1) Exenatide 2 mg QW + placebo (2) Dapagliflozin 10 mg once daily + placebo	104 weeks	Change in HbA1c from baseline to week 104; exploratory efficacy and safety outcomes including FPG, 2-h PPG, body weight, and systolic BP
Cannon et al., [15] (2020)	Multicenter, international	Randomized, double-blind, placebo-controlled	8246	Patients with type 2 diabetes and atherosclerotic cardiovascular disease	Ertugliflozin 5 mg or 15 mg once daily	Placebo	Mean 3.5 years	Major adverse cardiovascular events (composite of death from cardiovascular causes, nonfatal myocardial infarction, or nonfatal stroke)
Cahn et al., [16] (2021)	Multinational	Post hoc analysis of RCT	17,160 (dapagliflozin vs placebo)	Adults with type 2 diabetes; baseline use of GLAs including metformin, sulphonylureas, DPP-4 inhibitors, GLP-1 RAs, insulin	Dapagliflozin	Placebo	NR	Composite of cardiovascular death and hospitalization for heart failure; major adverse cardiovascular events; renal-specific outcomes
Blonde et al., [17] (2020)	Multinational	Parallel, double-blind RCT	303 (202 liraglutide, 100 placebo)	Patients with type 2 diabetes on stable SGLT2i ± metformin; HbA1c 7.0%–9.5%; BMI ≥20 kg/m²	Liraglutide 1.8 mg/day + SGLT2i ± metformin	Placebo + SGLT2i ± metformin	26 weeks	Change in HbA1c from baseline
Vernstrøm et al., [18] (2024)	Not specified	Randomized, parallel-group	120 (4 groups, n=30 each)	Adults with Type 2 Diabetes	Empagliflozin + Semaglutide (dose not specified)	Placebo, Empagliflozin alone, Semaglutide alone	32 weeks	Change in arterial stiffness (carotid-femoral pulse wave velocity), kidney oxygenation
Sivalingam et al., [19] (2024)	Denmark	Randomized, placebo-controlled, double-blind, parallel	60	Individuals with type 2 diabetes and albuminuria; all on renin-angiotensin system inhibition	Semaglutide 1 mg once weekly + Empagliflozin 25 mg once daily	Placebo 1 mg once weekly + Empagliflozin 25 mg once daily	26 weeks	Change in UACR
Lin et al., [20] (2024)	China	Randomized, double-blinded, parallel-group, active-controlled trial	105	Overweight/obese patients with NAFLD and T2DM	Semaglutide + Empagliflozin (dose not specified)	Semaglutide alone / Empagliflozin alone (dose not specified)	52 weeks	Change from baseline to week 52 in controlled attenuation parameter, free fatty acid, and glucagon

The study designs included post-hoc analyses of major RCTs [12,16], randomised, double-blind, placebo-controlled trials [13,15,17-19], and active-controlled trials [14,20]. The populations consistently focused on adults with type 2 diabetes, with most studies enrolling patients with established cardiovascular disease, high cardiovascular risk, or other comorbidities such as renal disease [12,13,15] or albuminuria [19]. The interventions examined were combinations of SGLT2i and GLP-1 RAs, including dapagliflozin with exenatide [14], liraglutide added to SGLT2i [17], and empagliflozin with semaglutide [18-20]. Comparators were predominantly placebo, often on top of standard care, or active monotherapy. Follow-up durations ranged from 26 weeks [17,19] to a mean of 3.5 years [15]. The primary outcomes were diverse, including MACE [12,13,15], changes in HbA1c [14,17], and various cardiometabolic and renal parameters [18,19].

Cardiovascular Outcomes

The impact of SGLT2i and GLP-1 RA combination therapy on hard cardiovascular endpoints was a key focus of several large studies. A post-hoc analysis by Neves et al. [12] demonstrated that GLP-1 RA therapy significantly reduced the risk of MACE (HR: 0.77) and heart failure hospitalisation (HR: 0.72) compared to placebo in patients not taking background SGLT2i. Crucially, the cardioprotective benefits were consistent in the subgroup of patients who were on background SGLT2i therapy, with no significant interaction (P for interaction = 0.95 for MACE), suggesting an additive effect of the two drug classes. Similarly, an exploratory analysis of the AMPLITUDE-O trial by Lam et al. [13] found that the GLP-1 RA efpeglenatide reduced MACE regardless of concomitant SGLT2i use, with hazard ratios of 0.74 without and 0.70 with SGLT2i.

Conversely, the cardiorenal benefits of SGLT2i were also shown to be maintained when used alongside GLP-1 RAs. Cahn et al. [16] reported that dapagliflozin reduced the composite of cardiovascular death and hospitalisation for heart failure, with the benefit being numerically greater in patients using baseline GLP-1 RAs (HR: 0.37) compared to non-users (HR: 0.86). Other CVOTs, such as that by Cannon et al. [15] for ertugliflozin, confirmed the cardiovascular safety of SGLT2i and their ability to reduce heart failure hospitalisations, though they were not specifically designed to test combination therapy. A summary of these key cardiometabolic outcomes is provided in Table 4.

**Table 4:** Cardiometabolic outcomes across included studies

Study (Year)	Major Adverse CV Events (MACE)	Heart Failure Hospitalization	Adverse Events (e.g., GI, Genital Infections)	Key Finding
Neves et al., [12] (2023)	HR: 0.77 (95% CI: 0.68–0.87) without SGLT2i; HR: 0.78 (95% CI: 0.49–1.24) with SGLT2i; P for interaction = 0.95	HR: 0.72 (95% CI: 0.55–0.92) without SGLT2i; HR: 0.34 (95% CI: 0.12–0.96) with SGLT2i; P for interaction = 0.18	No difference in adverse events; effect not modified by SGLT2 inhibitor use	GLP-1 RAs reduced MACE and heart failure hospitalization consistently, regardless of background SGLT2 inhibitor use, suggesting additive cardioprotective benefit of combination therapy.
Lam et al., [13] (2022)	Reduced MACE (HR 0.74 [0.58–0.94] without SGLT2i; 0.70 [0.37–1.30] with SGLT2i)	Reduction consistent with MACE outcomes	No difference in adverse events by SGLT2i use; typical GI side effects noted	Combination therapy (GLP-1 RA + SGLT2i) showed additive cardiometabolic and renal benefits without added safety concerns. Efficacy and safety were independent of concurrent SGLT2 inhibitor use.
Jabbour et al., [14] (2020)	NR	NR	Well tolerated; no major hypoglycemia or unexpected AEs	Combination therapy showed greater and sustained HbA1c, weight, and BP reductions over 104 weeks with good safety profile
Cannon et al., [15] (2020)	11.9% ertugliflozin vs 11.9% placebo; HR 0.97 (95.6% CI, 0.85–1.11)	8.1% ertugliflozin vs 9.1% placebo; HR 0.88 (95.8% CI, 0.75–1.03)	Amputations: 2.0–2.1% vs 1.6% placebo; other AEs not reported	Noninferior to placebo for MACE; trend toward reduced heart failure hospitalization
Cahn et al., [16] (2021)	HR 0.93 (95% CI 0.84–1.03), no significant interaction by baseline GLA	Reduced CVD + HHF, HR 0.37 [0.18, 0.78] in baseline GLP‑1 RA users vs 0.86 [0.75, 0.98] in non-users	NR	Dapagliflozin reduced heart failure hospitalization and composite CV death regardless of baseline GLA, with greater benefit in baseline GLP‑1 RA users; cardiorenal benefits consistent across baseline therapies.
Blonde et al., [17] (2020)	NR	NR	66.3% vs 47.0% ≥1 TEAE	Liraglutide added to SGLT2i ± metformin improved HbA1c significantly; weight loss modest; no unexpected safety findings
Vernstrøm et al., [18] (2024)	NR	NR	NR	Combination therapy improved glycaemic control and significantly reduced 24-h systolic BP without increasing hypoglycaemia; arterial stiffness unchanged
Sivalingam et al., [19] (2024)	NR	NR	NR	Semaglutide added to empagliflozin improved HbA1c but did not change body weight, BP, or eGFR; CV outcomes and adverse events not reported
Lin et al., [20] (2024)	NR	NR	NR	Designed to assess synergistic cardiometabolic benefits and safety; results pending

Glycaemic Control, Weight, and Blood Pressure

Combination therapy consistently demonstrated superior glycaemic control and ancillary benefits compared to monotherapy or placebo. The DURATION-8 trial by Jabbour et al. [14] showed that the combination of exenatide and dapagliflozin led to significantly greater and sustained reductions in HbA1c, body weight, and systolic blood pressure over 104 weeks compared to either agent alone. Similarly, Blonde et al. [17] found that adding liraglutide to patients inadequately controlled on SGLT2i significantly improved HbA1c, with modest additional weight loss. Vernstrøm et al. [18] reported that the combination of empagliflozin and semaglutide improved glycaemic control and resulted in a significant reduction in 24-hour systolic blood pressure compared to either drug alone, without increasing the risk of hypoglycaemia.

Renal and Other Cardiometabolic Parameters

The effects on renal and other vascular parameters were explored in smaller, dedicated studies. Sivalingam et al. [19] focused specifically on albuminuria, finding that while the addition of semaglutide to empagliflozin further improved HbA1c, it did not lead to significant changes in body weight, blood pressure, or estimated glomerular filtration rate (eGFR) over 26 weeks, and its effect on albuminuria was the primary focus. Vernstrøm et al. [18] investigated vascular function and found that despite improvements in blood pressure and glycaemia, the combination therapy did not alter arterial stiffness (measured by carotid-femoral pulse wave velocity) more than the individual drugs. One ongoing trial by Lin et al. [20] is specifically designed to assess the synergistic effects of semaglutide and empagliflozin on cardiometabolic parameters, including liver fat content in patients with NAFLD and T2DM, with results pending.

Safety and Tolerability

The safety profile of combination therapy was generally consistent with the known profiles of the individual drug classes. Studies consistently reported no significant difference in the overall rate of adverse events or serious adverse events between combination therapy and comparator groups [12-14]. The combination was described as "well-tolerated" [14] with "no unexpected safety findings" [17]. The most commonly reported adverse events were gastrointestinal in nature, typical of GLP-1 RA therapy [13,17]. There was no evidence that combining an SGLT2i with a GLP-1 RA increased the risk of specific adverse events like genital infections or hypoglycaemia beyond what is expected from each drug class alone [12,14,18].

Risk of Bias in Included Studies

The methodological quality of the included studies, as assessed by the Cochrane Risk of Bias tool (RoB 2), was variable, with the overall risk judged as low for the majority of primary randomised controlled trials [12,14,15,17-20]. However, two studies [13,16] were deemed to have a high overall risk of bias. These two studies were exploratory or post-hoc analyses of larger trials, which introduced a high risk of bias due to deviations from the intended interventions, as the compared groups (e.g., with or without concomitant SGLT2 inhibitor use) were not randomised. Furthermore, these studies [13,16] raised some concerns in the domains of the randomisation process and the selection of the reported result, given their non-pre-specified, subgroup nature. In contrast, the remaining seven studies, which reported on their pre-specified primary outcomes, demonstrated a low risk of bias across all five domains, including the randomisation process, deviations from intended interventions, missing outcome data, measurement of the outcome, and selection of the reported result (Table 5).

**Table 5:** Risk of bias assessment using Cochrane RoB 2 tool

Study (Year)	D1: Randomization Process	D2: Deviations from Intended Interventions	D3: Missing Outcome Data	D4: Measurement of the Outcome	D5: Selection of Reported Result	Overall Bias
Neves et al., [12] (2023)	Low	Low	Low	Low	Low	Low
Lam et al., [13] (2022)	Some Concerns	High	Low	Low	Some Concerns	High
Jabbour et al., [14] (2020)	Low	Low	Low	Low	Low	Low
Cannon et al., [15] (2020)	Low	Low	Low	Low	Low	Low
Cahn et al., [16] (2021)	Some Concerns	High	Low	Low	Some Concerns	High
Blonde et al., [17] (2020)	Low	Low	Low	Low	Low	Low
Vernstrøm et al., [18] (2024)	Low	Low	Low	Low	Low	Low
Sivalingam et al., [19] (2024)	Low	Low	Low	Low	Low	Low
Lin et al., [20] (2024)	Low	Low	Low	Low	Low	Low

Discussion

This systematic review synthesises evidence from nine studies [12-20] to evaluate the cardiometabolic benefits and risks of combining SGLT2i and GLP-1 RAs in patients with type 2 diabetes. The findings collectively suggest that this combination therapy offers additive or synergistic benefits for cardiovascular risk reduction, glycaemic control, and weight management, without a concomitant increase in adverse events beyond the known safety profiles of the individual drug classes. The evidence base, however, is nuanced, derived primarily from subgroup analyses of CVOTs and smaller mechanistic studies, which informs both the strength and the limitations of the current conclusions.

The most compelling finding from this review pertains to cardiovascular outcomes. The post-hoc analyses by Neves et al. [12] and Lam et al. [13] provide robust, albeit exploratory, evidence that the cardioprotective effects of GLP-1 RAs are preserved and potentially additive to those of SGLT2is. The consistent reduction in MACE and heart failure hospitalisation observed with GLP-1 RA therapy, regardless of background SGLT2i use, indicates that these two drug classes act through complementary, rather than redundant, pathways. This is a significant observation, as it suggests that intensification of therapy with a GLP-1 RA in a patient already on an SGLT2i, a common clinical scenario, confers additional cardiovascular benefit. Conversely, the analysis by Cahn et al. [16] reinforces this concept from the SGLT2i perspective, demonstrating that the profound reduction in heart failure hospitalisations and cardiovascular death with dapagliflozin is not attenuated by concomitant GLP-1 RA use; in fact, a numerically greater benefit was observed in this subgroup. This bidirectional consistency strongly supports the notion of additive cardioprotection. These findings align with the pathophysiological understanding that GLP-1 RAs predominantly exert anti-atherosclerotic effects through direct vascular and anti-inflammatory mechanisms, while SGLT2 primarily confer benefits via haemodynamic, metabolic, and direct cardiac effects. Our results are consistent with a growing body of real-world evidence and meta-analyses, such as that by Zelniker et al. [21], which established the distinct yet complementary cardiovascular benefits of SGLT2is and GLP-1 RAs at a class level. Similarly, a network meta-analysis by Palmer et al. [22] concluded that combinations of these agents are likely to provide the greatest reduction in cardiovascular risk, a hypothesis our review substantiates with direct, though indirect, comparative evidence.

Beyond hard cardiovascular endpoints, the combination therapy demonstrates a superior impact on key cardiometabolic parameters. The results from Jabbour et al. [14] and Blonde et al. [17] clearly show that combining a GLP-1 RA with an SGLT2i leads to significantly greater and more sustained reductions in HbA1c compared to either agent alone. This is clinically paramount for patients failing to achieve glycaemic targets on monotherapy, offering a potent oral-injectable or dual-injectable regimen that avoids the weight gain and hypoglycaemia risks associated with insulin or sulfonylureas. Furthermore, the synergistic effect on body weight is a standout advantage. The DURATION-8 trial [14] showed substantial weight loss with the exenatide-dapagliflozin combination, a finding that is physiologically plausible given the distinct anorexigenic and caloric excretory mechanisms of GLP-1 RAs and SGLT2is, respectively. This aligns with the findings of Frías et al. [23] in the DURATION-8 population, who reported superior and sustained weight reduction with the combination. The additional weight loss observed by Blonde et al. [17] with liraglutide added to SGLT2i, though modest, further confirms the additive effect. The significant reduction in systolic blood pressure reported by both Jabbour et al. [14] and Vernstrøm et al. [18] provides another compelling rationale for the combination, particularly in a population where hypertension is a common comorbidity and a major driver of cardiovascular and renal risk. This blood pressure-lowering effect is likely multifactorial, resulting from the natriuretic and osmotic diuretic effects of SGLT2is combined with the potential vasodilatory and weight-loss effects of GLP-1 RAs.

However, the review also highlights areas where the combination may not yield synergistic benefits. The study by Vernstrøm et al. [18] is particularly insightful, as it found that despite improvements in glycaemia and 24-hour systolic blood pressure, the combination of empagliflozin and semaglutide did not lead to a greater improvement in arterial stiffness (measured by pulse wave velocity) than either drug alone. This suggests that the potent functional and metabolic benefits of the combination may not directly translate to all measures of vascular pathology, or that a longer intervention period is required to observe structural vascular changes. This finding is consistent with the work of Ikonomidis et al. [24], who showed that while GLP-1 RAs can improve arterial stiffness, the effects can be variable and may depend on baseline vascular health and treatment duration. Similarly, the study by Sivalingam et al. [19] reported that adding semaglutide to empagliflozin improved HbA1c but did not significantly alter body weight, blood pressure, or eGFR over 26 weeks, indicating that the benefits may be more specific to glycaemic control in certain populations and that the renal benefits, particularly on albuminuria, which was the primary outcome, require further elucidation. This contrasts with the marked renal protective effects seen when each drug class is used individually, as demonstrated in trials like LEADER for liraglutide [25] and CREDENCE for canagliflozin [26], and suggests that the renal interaction may be complex and not simply additive.

The reassuring safety profile of the combination therapy is a critical finding for clinical practice. Across the included studies [12-14,17,18], there was no signal for new or unexpected safety concerns. The adverse event profile was a predictable amalgamation of the known effects of each class: an increased incidence of gastrointestinal events (e.g., nausea, diarrhoea) attributable to GLP-1 RAs and a potential for genital mycotic infections associated with SGLT2is. Importantly, there was no evidence of a pharmacodynamic interaction that heightened the risk of these events or of serious adverse events like severe hypoglycaemia. This safety profile is a significant advantage over older combination regimens and is consistent with the established safety of these drug classes. For instance, a large retrospective cohort study by Dawwas et al. [27] found that the combination of SGLT2is and GLP-1 RAs was not associated with an increased risk of serious adverse events compared to other dual therapy combinations. The tolerability of this combination is a key factor that will influence its adoption in clinical practice, as the benefits are not offset by significant safety drawbacks.

A notable strength of the evidence base is the methodological rigour of the primary RCTs included, as reflected in the generally low risk of bias for studies like Jabbour et al. [14], Cannon et al. [15], and others [17-19]. However, the review is necessarily shaped by the limitations of the available literature. The most significant evidence for cardiovascular outcomes comes from post-hoc or exploratory analyses [12,13,16], which, while highly informative, inherit a high risk of bias as the compared subgroups were not randomised. These analyses generate a powerful hypothesis of additive benefit but must be confirmed by prospectively designed trials. The ongoing trial by Lin et al. [20] is a step in this direction, specifically designed to assess the synergistic effects on nonalcoholic fatty liver disease (NAFLD), and its results are eagerly awaited. Furthermore, the populations in the included studies were predominantly high-risk, with established cardiovascular or renal disease. While this is precisely the population that stands to gain the most from combination therapy, the generalisability of these findings to lower-risk individuals with type 2 diabetes remains to be established. Finally, the follow-up durations of the smaller mechanistic studies [17-19] were relatively short (26-32 weeks), limiting our understanding of the long-term durability of the glycaemic, weight, and vascular benefits observed.

Limitations

This systematic review has several limitations. First, the number of included studies is modest (n=9), and the evidence for hard cardiovascular outcomes is primarily derived from post-hoc analyses, which are hypothesis-generating but not conclusive. Second, there was significant clinical and methodological heterogeneity among the studies, particularly in terms of study designs (CVOTs vs. mechanistic studies), specific drug combinations investigated, comparator groups, and primary outcomes, precluding a meta-analysis. Third, the follow-up duration was insufficient in several studies to assess long-term outcomes and the sustainability of benefits. Fourth, the search was limited to published literature, and publication bias cannot be ruled out. Finally, the generalisability of findings may be limited to patients with established cardiovascular disease or high cardiovascular risk, as this was the predominant population in the larger trials.

## Conclusions

The combination of SGLT2 inhibitors and GLP-1 receptor agonists in patients with type 2 diabetes offers additive benefits for cardiovascular risk reduction, glycaemic control, weight management, and blood pressure, without introducing new safety concerns beyond the established profiles of each drug class. The complementary mechanisms of action of these two classes create a synergistic therapeutic profile that addresses multiple facets of cardiometabolic disease. While the current evidence is strongly supportive, it is primarily grounded in exploratory analyses of major trials. Therefore, large-scale, prospective, randomised controlled trials specifically designed to evaluate the efficacy and safety of this combination are warranted to provide definitive evidence and guide its optimal implementation in clinical practice. Until then, the existing data robustly supported the use of this combination as a potent therapeutic strategy for high-risk patients with type 2 diabetes who require intensified management to achieve comprehensive cardiometabolic protection.
